# Immunomodulation by vitamin D 

**DOI:** 10.5414/ALX1430E

**Published:** 2018-09-01

**Authors:** M. Worm, G. Heine, A. Radbruch

**Affiliations:** Allergie-Centrum Charité, Klinik für Dermatologie und allergologie, Charité Campus Mitte – Universitätsmedizin Berlin, Germany

**Keywords:** Key words vitamin D, calcitriol, immunomodulation, B cells

## Abstract

Vitamin D exerts several immunological functions in addition to its homeostatic functions on calcium and bone metabolism. Current data show that relative vitamin D deficiency (< 75 nmol/l 25-hydroxyvitamin D) as well as acquired seasonal vitamin D deficiency (< 50 nmol/l) are frequent in Germany. As confirmed by our own data, UV exposure plays a major role for maintenance of vitamin D status, e.g., in patients with UV-triggered diseases, vitamin D deficiency is more frequent, even throughout the year. The beneficial impact of vitamin D on immune functions is highlighted by epidemiologic, genetic, and experimental evidence. In the past years, numerous publications have presented associations between vitamin D deficiency, on the one hand, and severity and prevalence of allergic asthma in children and adults, on the other hand.

**German version published in Allergologie, Vol. 34, No. 11/2011, pp. 538-542**

## Introduction 

**Figure 1.**
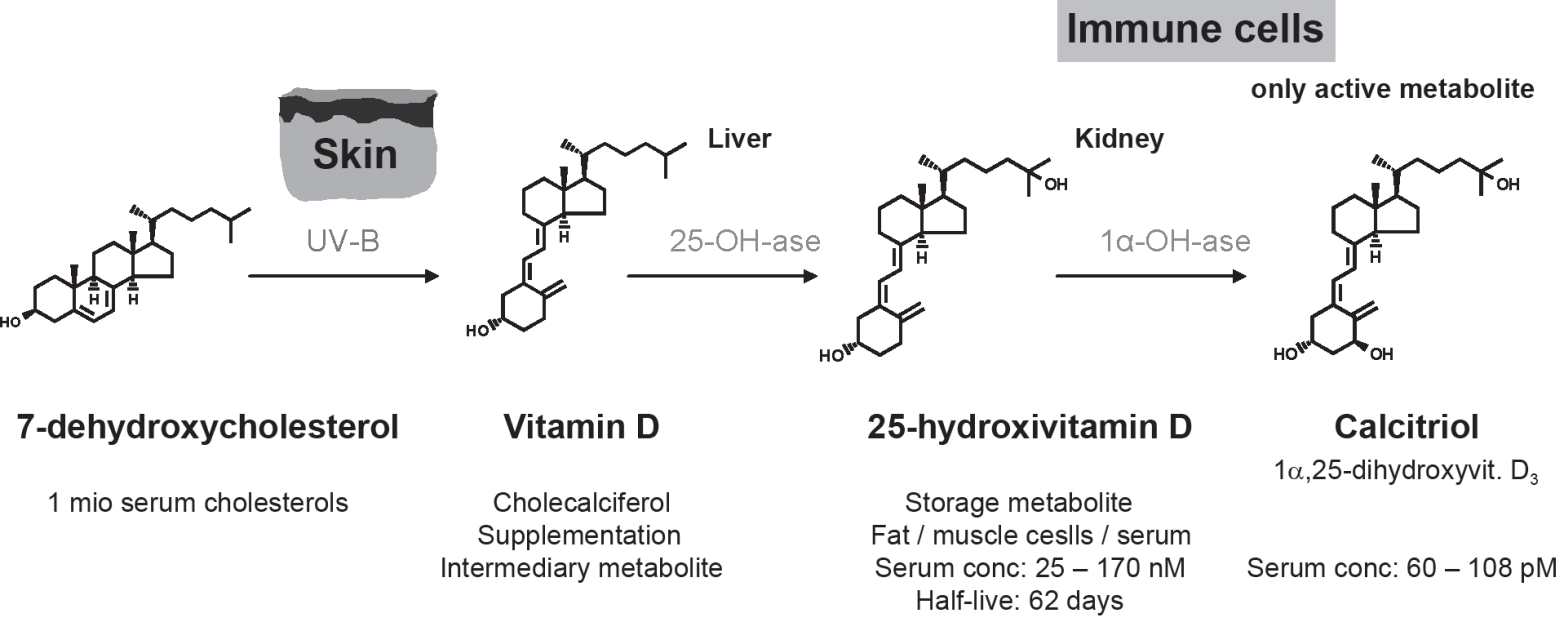
Vitamin D is a secosteroid hormone.

**Figure 2.**
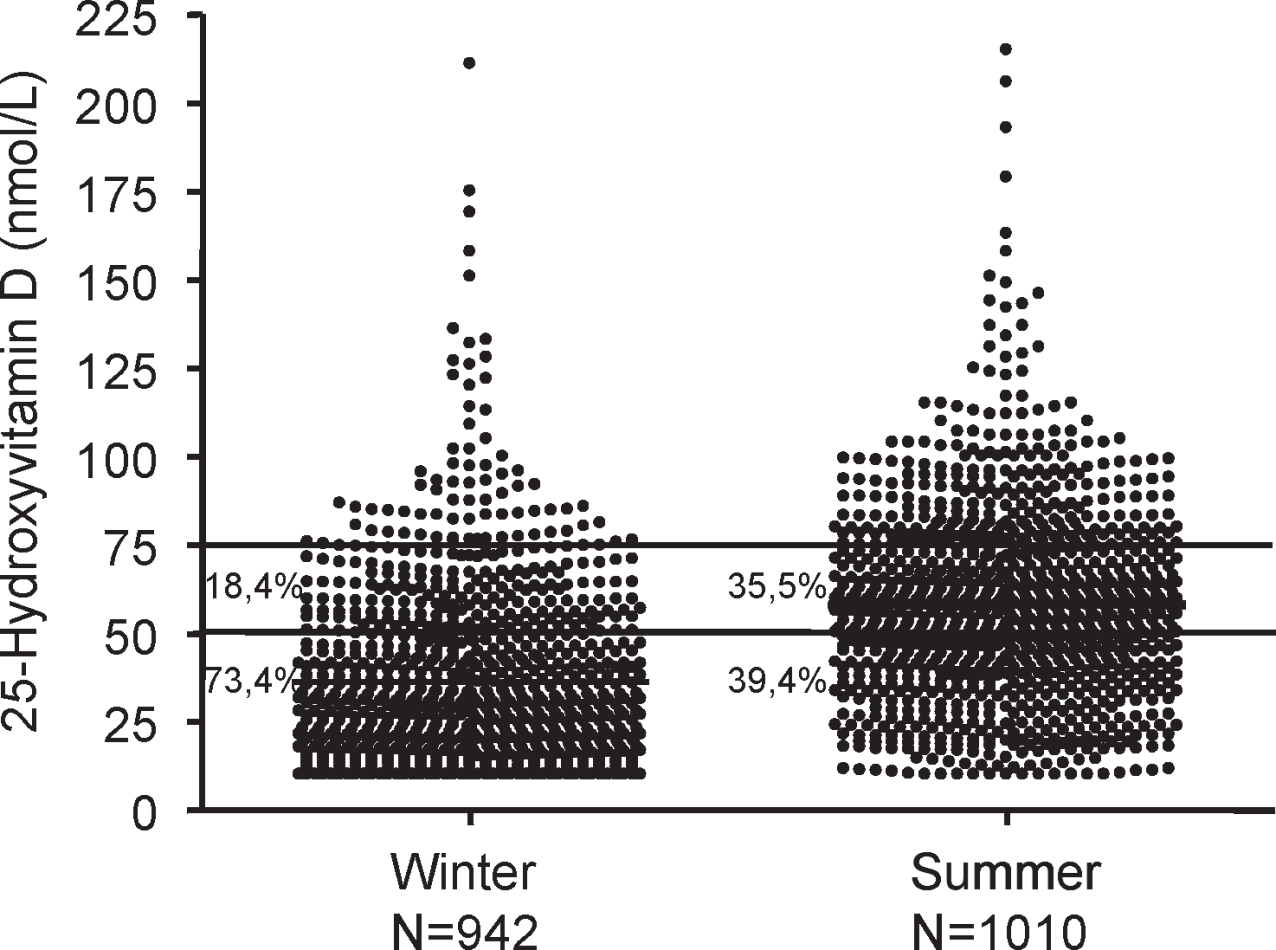
Relative vitamin D deficiency is frequent nowadays.

Vitamin D is a steroid hormone produced by the human body from 7-dehydrocholesterol when exposure to UV radiation is present [20]. After enzymatic hydroxylation, it is available in its active form 1α25-hydroxyvitamin D (also called calcitriol) (Figure 1) [20]. It is essential for bone homeostasis as it promotes the resorption of nutritional calcium in the intestine and the kidney [19]. Although today the nutritional situation in the general population is very good, a relative vitamin D deficiency can occur, particularly in winter. A report by Hinzpeter et al. [18], for example, shows that in more than 80% of Germans the daily vitamin D intake with nutrition is below the recommended dose of 5 mg/day. Accordingly, more than 55% of Germans have a relative vitamin D deficiency (serum concentration of 25-hydroxyvitamin D < 50 nmol/l) [18]. Current research shows an increasing frequency of relative vitamin D deficiency over the past decade despite the widespread use of vitamin D supplementation [27]. It is very probable that an insufficient UV exposition due to indoor working and increasing use of sunscreens plays an important role [27]. Our own investigation in more than 1,900 patients from Berlin [16] showed that in summer a relative vitamin D deficiency was present in 39.4% of the investigated patients and in winter, where there is only little UV radiation, this rate was as high as 73.4% (Figure 2) [16]. In the human body, vitamin D is mainly produced by UV radiation biosynthesis [21]. In autoimmune patients with cutaneous lupus erythematosus, who have to avoid UV exposure due to its disease-promoting effects, a relative vitamin D deficiency is present in 85.7% of patients, even in the summer months, and in winter this rate increases to 97.1% [16]. These data underline that a relative vitamin D deficiency is frequent in Germany, particularly in individuals whose daily exposure to UV radiation is limited due to various reasons. 

## Vitamin D and immune system 

**Figure 3.**
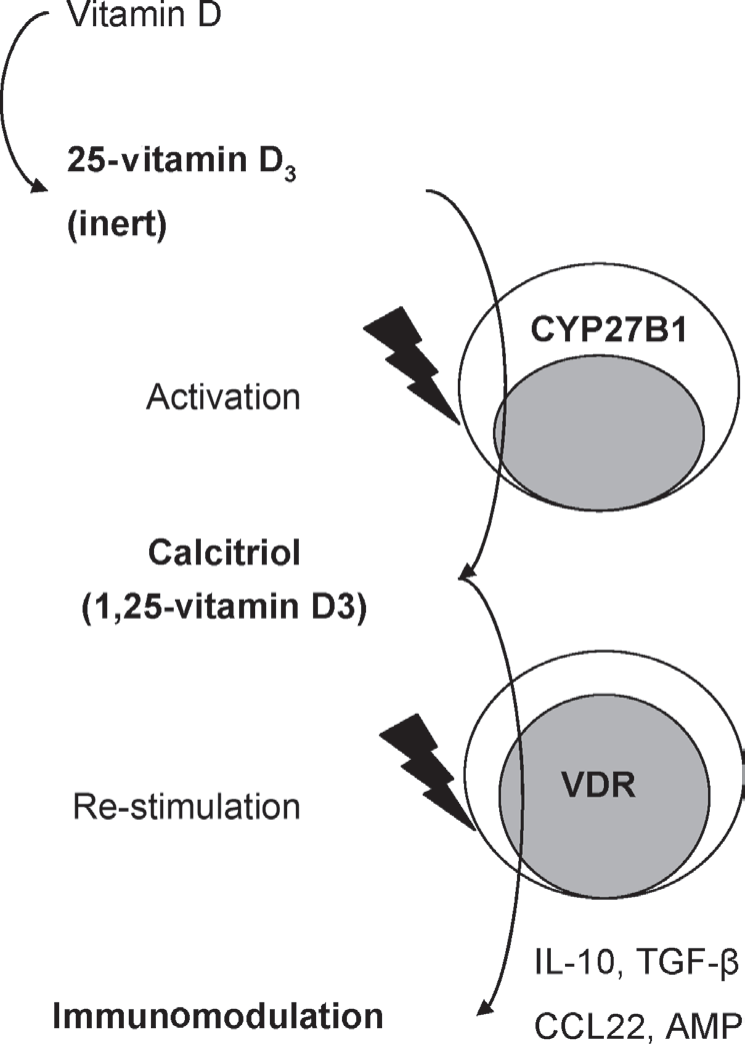
Autocrine calcitriol synthesis and effects in immune cells.

Extensive investigation has suggested an immunomodulatory effect of vitamin D [2, 5, 13, 31]. It also seems to play a role in allergic diseases like allergic bronchial asthma [24, 26]. Epidemiological data show that in the northern parts of the USA a higher prevalence of allergic diseases is present than in the southern parts where there is more UV radiation [7]. Numerous reports suggest a relationship between vitamin D deficiency and the prevalence of allergic diseases as well as a relationship with the severity of allergic asthma and a hyperreactive bronchial system in children, adolescents [6, 9], and adults [25]. Maternal vitamin D supplementation was shown to reduce the prevalence of juvenile wheezing in 3-year-old children [8]. However, the existing data is not unambiguous [22, 40]. It is thought that vitamin D deficiency increases glucocorticoid resistance, as an increased glucocorticoid consumption in the body and a reduced pulmonary function could be shown in vitamin D-deficient children and adults [37, 38]. Furthermore, gene analyses support the hypothesis that vitamin D plays a role in the pathogenesis of allergic asthma. For example, point mutations in the vitamin D receptor gene with a possible loss of function were found in two independent cohorts in the USA [33, 34], but not in a comparable German cohort [39]. The therapeutic use of vitamin D is limited by its hypercalcemic side effects. Nevertheless, the administration of 2 × 0.25 mg calcitriol p.o. for 7 days resulted in an improvement of glucocorticoid resistance in asthma patients, probably due to the induction of IL-10 producing regulatory Tr1 cells [42]. In parallel, increased IL-10 serum concentrations were measured in patients with congestive heart failure after supplementation with 2,000 IU of vitamin D [36]. Also, in patients with multiple sclerosis, an increase of the TGF-β production by CD4 T-helper cells could be detected after the administration of 1,000 IU/day of vitamin D [28], and after high-dose vitamin D supplementation even positive effects on disease severity could be demonstrated [23]. Vitamin D can act directly on immune cells, like antigen-presenting cells, but also on T and B lymphocytes [1, 29, 31]. Among the numerous possible effects are an altered, pro-tolerogenic activation of dendritic cells [3] or the regulation of the humoral immune response [12]. Our own investigation showed that vitamin D, on the one hand, inhibits IgE production, e.g., in B cells [14] and, on the other hand, induces the tolerogenic cytokine IL-10 (Figure 3) [15]. Interestingly, B cells can independently produce active calcitriol [15] so that immunoregulatory effects on B cells can also be obtained by supplementation of the precursor form. In a first clinical investigation, we could demonstrate that the supplementation of 2,000 IU of vitamin D in winter significantly increases the vitamin D concentration, and that this does not negatively (i.e., in the sense of an immunosuppression) influence a robust recall response, as measured by peripheral immune responses at the T cell and B cell level [17]. Our further investigation showed that even a supplementation of up to 8,000 IU during the winter months results in the normalization of the vitamin D level without having side effects, while immune cells are specifically modulated (Drozdenko et al., unpublished data). 

## Vitamin D analogs 

Due to its hypercalcemic effects, the therapeutic use of vitamin D is limited [2, 29]. Thus, synthetic derivatives have been developed in which the calcium-mobilizing effects are dissociated from the immunologic effects. These kinds of calcitriol analogs are already being used in clinical practice for the topic therapy of psoriasis vulgaris (a chronic inflammatory skin disease) [29]. The exact molecular mechanism of these derivatives has not yet been fully elucidated, but cell-specific effects have been described. The calcitriol analog ZK191784, for example, does not have calcium-resorbing effects on intestinal mucosal cells [32], but it has anti-inflammatory effects on T cells [43]. Our own investigation shows that in a murine model the systemic IgE response could be reduced by systemic treatment with a low-calcemic calcitriol analog [12]. Even very structurally different derivatives can be immunologically effective; the calcitriol derivative BXL-219, for example, prevents experimentally induced Type 1 diabetes [4], and another derivative, ZK156979, inhibits experimental colitis [11]. In conclusion, these data underline the complex effect of vitamin D receptors on the immune response. Future data will demonstrate the therapeutic benefit of derivatives for the treatment of immune-mediated diseases in humans. 

## Nuclear hormone receptor ligands control IgE response 

**Figure 4.**
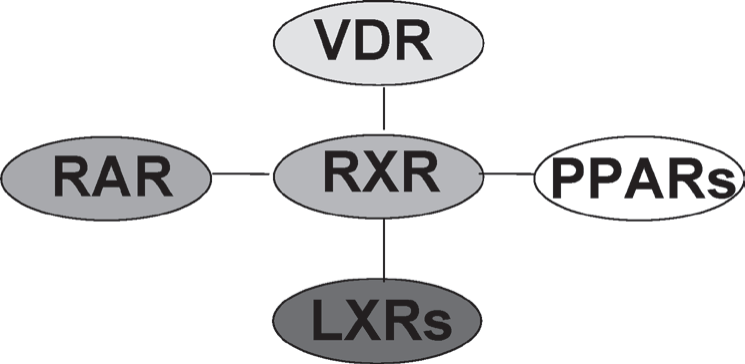
Family of nuclear hormone receptors.

**Figure 5.**
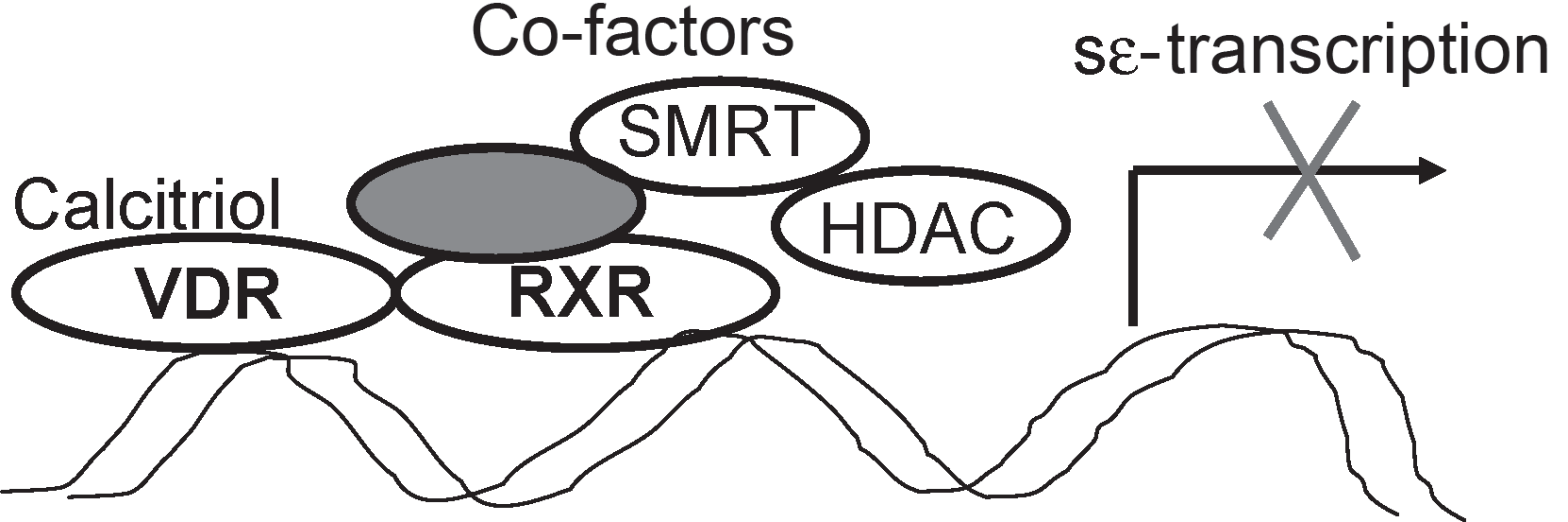
Inhibition of IgE isotype class switching by interaction of the vitamin D receptor in the e-germline promoter.

Vitamin D binds to its receptor (VDR) in the cytosol, and after translocation of this calcitriol-VDR complex into the cell nucleus, numerous genes are activated or inhibited [13]. In earlier investigations we could show that vitamin D is not the only nuclear hormone receptor ligand inhibiting IgE production. Other members of this family have the same effect: retinoids [41], liver X receptor ligands [14, 30], and PPARs [10, 35] (Figure 4). Recently, we could confirm these in-vitro findings in the mouse model [12]. This may be the basis for an innovative therapeutic approach to use vitamin D in the treatment of allergic diseases. The fact that vitamin D inhibits the transcription factor NFκB, which is essential for the switching of the IgE isotype class, seems to play an important role in the mechanism [14]. Our further investigation demonstrated that the vitamin D receptor can inhibit the switching of the isotype class to IgE directly in the IgE switch promoter (e-germline promoter) by recruiting inhibitory molecules (Figure 5) [30]. Future studies will have to clarify whether this leads to a stable or unstable modulation of the allergic immune response, and how this could be used for the prevention or treatment of allergic diseases. 

## Conclusion and perspectives 

Vitamin D deficiency is very common in higher latitudes and can be relevant for the development of osteoporosis and immunologic diseases as vitamin D can have numerous protective and immunomodulatory effects. Vitamin D or its precursors might be beneficial for the prevention and therapy of diseases with a modified immune response, and this potential should be investigated in future clinical studies. 

## References

[1] Adams JS Chen H Chun R Ren S Wu S Gacad M Nguyen L Ride J Liu P Modlin R Hewison M Substrate and enzyme trafficking as a means of regulating 1,25-dihydroxyvitamin D synthesis and action: the human innate immune response. J Bone Miner Res. 2007; 22: V20–V24. 18290716 10.1359/jbmr.07s214

[2] Adams JS Hewison M Unexpected actions of vitamin D: new perspectives on the regulation of innate and adaptive immunity. Nat Clin Pract Endocrinol Metab. 2008; 4: 80–90. 18212810 10.1038/ncpendmet0716 PMC2678245

[3] Adorini L Tolerogenic dendritic cells induced by vitamin D receptor ligands enhance regulatory T cells inhibiting autoimmune diabetes. Ann N Y Acad Sci. 2003; 987: 258–261. 12727648 10.1111/j.1749-6632.2003.tb06057.x

[4] Adorini L Amuchastegui S Corsiero E Laverny G Le Meur T Penna G Vitamin D receptor agonists as anti-inflammatory agents. Expert Rev Clin Immunol. 2007; 3: 477–489. 20477154 10.1586/1744666X.3.4.477

[5] Adorini L Penna G Control of autoimmune diseases by the vitamin D endocrine system. Nat Clin Pract Rheumatol. 2008; 4: 404–412. 18594491 10.1038/ncprheum0855

[6] Brehm JM Celedón JC Soto-Quiros ME Avila L Hunninghake GM Forno E Laskey D Sylvia JS Hollis BW Weiss ST Litonjua AA Serum vitamin D levels and markers of severity of childhood asthma in Costa Rica. Am J Respir Crit Care Med. 2009; 179: 765–771. 19179486 10.1164/rccm.200808-1361OC PMC2675563

[7] Camargo CA Clark S Kaplan MS Lieberman P Wood RA Regional differences in EpiPen prescriptions in the United States: the potential role of vitamin D. J Allergy Clin Immunol. 2007; 120: 131–136. 17559916 10.1016/j.jaci.2007.03.049

[8] Camargo CA Rifas-Shiman SL Litonjua AA Rich-Edwards JW Weiss ST Gold DR Kleinman K Gillman MW Maternal intake of vitamin D during pregnancy and risk of recurrent wheeze in children at 3 y of age. Am J Clin Nutr. 2007; 85: 788–795. 17344501 10.1093/ajcn/85.3.788 PMC4406411

[9] Chinellato I Piazza M Sandri M Peroni DG Cardinale F Piacentini GL Boner AL Serum vitamin D levels and exercise-induced bronchoconstriction in children with asthma. Eur Respir J. 2011; 37: 1366–1370. 21071468 10.1183/09031936.00044710

[10] Dahten A Koch C Ernst D Schnöller C Hartmann S Worm M Systemic PPARgamma ligation inhibits allergic immune response in the skin. J Invest Dermatol. 2008; 128: 2211–2218. 18401424 10.1038/jid.2008.84

[11] Daniel C Schlauch T Zügel U Steinmeyer A Radeke HH Steinhilber D Stein J 22-ene-25-oxa-vitamin D: a new vitamin D analogue with profound immunosuppressive capacities. Eur J Clin Invest. 2005; 35: 343–349. 15860047 10.1111/j.1365-2362.2005.01492.x

[12] Hartmann B Heine G Babina M Steinmeyer A Zugel U Radbruch A Targeting the vitamin D receptor inhibits the B cell-dependent allergic immune response. Allergy. 2010; epub ahead of print. 10.1111/j.1398-9995.2010.02513.x 21121929

[13] Hayes CE Nashold FE Spach KM Pedersen LB The immunological functions of the vitamin D endocrine system. Cell Mol Biol. 2003; 49: 277–300. 12887108

[14] Heine G Anton K Henz BM Worm M 1alpha,25-dihydroxyvitamin D3 inhibits anti-CD40 plus IL-4-mediated IgE production in vitro. Eur J Immunol. 2002; 32: 3395–3404. 12432570 10.1002/1521-4141(200212)32:12<3395::AID-IMMU3395>3.0.CO;2-#

[15] Heine G Niesner U Chang HD Steinmeyer A Zügel U Zuberbier T Radbruch A Worm M 1,25-dihydroxyvitamin D(3) promotes IL-10 production in human B cells. Eur J Immunol. 2008; 38: 2210–2218. 18651709 10.1002/eji.200838216

[16] Heine G Lahl A Müller C Worm M Vitamin D deficiency in patients with cutaneous lupus erythematosus is prevalent throughout the year. Br J Dermatol. 2010; 163: 863–865. 20662833 10.1111/j.1365-2133.2010.09948.x

[17] Heine G Drozdenko G Lahl A Unterwalder N Mei H Volk H-D Dörner T Radbruch A Worm M Efficient tetanus toxoid immunization on vitamin D supplementation. Eur J Clin Nutr. 2011; 65: 329–334. 21224870 10.1038/ejcn.2010.276

[18] Hintzpeter B Mensink GB Thierfelder W Müller MJ Scheidt-Nave C Vitamin D status and health correlates among German adults. Eur J Clin Nutr. 2008; 62: 1079–1089. 17538533 10.1038/sj.ejcn.1602825

[19] Holick MF Resurrection of vitamin D deficiency and rickets. J Clin Invest. 2006; 116: 2062–2072. 16886050 10.1172/JCI29449 PMC1523417

[20] Holick MF Vitamin D deficiency. N Engl J Med. 2007; 357: 266–281. 17634462 10.1056/NEJMra070553

[21] Hollis BW Circulating 25-hydroxyvitamin D levels indicative of vitamin D sufficiency: implications for establishing a new effective dietary intake recommendation for vitamin D. J Nutr. 2005; 135: 317–322. 15671234 10.1093/jn/135.2.317

[22] Hyppönen E Sovio U Wjst M Patel S Pekkanen J Hartikainen AL Järvelinb MR Infant vitamin d supplementation and allergic conditions in adulthood: northern Finland birth cohort 1966. Ann N Y Acad Sci. 2004; 1037: 84–95. 15699498 10.1196/annals.1337.013

[23] Kimball SM Ursell MR O’Connor P Vieth R Safety of vitamin D3 in adults with multiple sclerosis. Am J Clin Nutr. 2007; 86: 645–651. 17823429 10.1093/ajcn/86.3.645

[24] Lange NE Litonjua A Hawrylowicz CM Weiss S Vitamin D, the immune system and asthma. Expert Rev Clin Immunol. 2009; 5: 693–702. 20161622 10.1586/eci.09.53 PMC2812815

[25] Li F Peng M Jiang L Sun Q Zhang K Lian F Litonjua AA Gao J Gao X Vitamin D deficiency is associated with decreased lung function in Chinese adults with asthma. Respiration. 2011; 81: 469–475. 21124013 10.1159/000322008 PMC3124457

[26] Litonjua AA Weiss ST Is vitamin D deficiency to blame for the asthma epidemic? J Allergy Clin Immunol. 2007; 120: 1031–1035. 17919705 10.1016/j.jaci.2007.08.028

[27] Looker AC Pfeiffer CM Lacher DA Schleicher RL Picciano MF Yetley EA Serum 25-hydroxyvitamin D status of the US population: 1988-1994 compared with 2000-2004. Am J Clin Nutr. 2008; 88: 1519–1527. 19064511 10.3945/ajcn.2008.26182 PMC2745830

[28] Mahon BD Gordon SA Cruz J Cosman F Cantorna MT Cytokine profile in patients with multiple sclerosis following vitamin D supplementation. J Neuroimmunol. 2003; 134: 128–132. 12507780 10.1016/s0165-5728(02)00396-x

[29] May E Asadullah K Zügel U Immunoregulation through 1,25-dihydroxyvitamin D3 and its analogs. Curr Drug Targets Inflamm Allergy. 2004; 3: 377–393. 15584887 10.2174/1568010042634596

[30] Milovanovic M Heine G Hallatschek W Opitz B Radbruch A Worm M Vitamin D receptor binds to the ε germline gene promoter and exhibits transrepressive activity. J Allergy Clin Immunol. 2010; 126: 1016–1023, 1023.e1-1023.e4.. 20926124 10.1016/j.jaci.2010.08.020

[31] Mora JR Iwata M von Andrian UH Vitamin effects on the immune system: vitamins A and D take centre stage. Nat Rev Immunol. 2008; 8: 685–698. 19172691 10.1038/nri2378 PMC2906676

[32] Nijenhuis T van der Eerden BCJ Zügel U Steinmeyer A Weinans H Hoenderop JGJ van Leeuwen JP Bindels RJ The novel vitamin D analog ZK191784 as an intestine-specific vitamin D antagonist. FASEB J. 2006; 20: 2171–2173. 17012263 10.1096/fj.05-5515fje

[33] Poon AH Laprise C Lemire M Montpetit A Sinnett D Schurr E Hudson TJ Association of vitamin D receptor genetic variants with susceptibility to asthma and atopy. Am J Respir Crit Care Med. 2004; 170: 967–973. 15282199 10.1164/rccm.200403-412OC

[34] Raby BA Lazarus R Silverman EK Lake S Lange C Wjst M Weiss ST Association of vitamin D receptor gene polymorphisms with childhood and adult asthma. Am J Respir Crit Care Med. 2004; 170: 1057–1065. 15282200 10.1164/rccm.200404-447OC

[35] Rühl R Dahten A Schweigert FJ Herz U Worm M Inhibition of IgE-production by peroxisome proliferator-activated receptor ligands. J Invest Dermatol. 2003; 121: 757–764. 14632193 10.1046/j.1523-1747.2003.12493.x

[36] Schleithoff SS Zittermann A Tenderich G Berthold HK Stehle P Koerfer R Vitamin D supplementation improves cytokine profiles in patients with congestive heart failure: a double-blind, randomized, placebo-controlled trial. Am J Clin Nutr. 2006; 83: 754–759. 16600924 10.1093/ajcn/83.4.754

[37] Searing DA Zhang Y Murphy JR Hauk PJ Goleva E Leung DY Decreased serum vitamin D levels in children with asthma are associated with increased corticosteroid use. J Allergy Clin Immunol. 2010; 125: 995–1000. 20381849 10.1016/j.jaci.2010.03.008 PMC2866800

[38] Sutherland ER Goleva E Jackson LP Stevens AD Leung DY Vitamin D levels, lung function, and steroid response in adult asthma. Am J Respir Crit Care Med. 2010; 181: 699–704. 20075384 10.1164/rccm.200911-1710OC PMC2868500

[39] Wjst M Variants in the vitamin D receptor gene and asthma. BMC Genet. 2005; 6:2. 15651992 10.1186/1471-2156-6-2 PMC546000

[40] Wjst M The vitamin D slant on allergy. Pediatr Allergy Immunol. 2006; 17: 477–483. 17014620 10.1111/j.1399-3038.2006.00456.x

[41] Worm M Krah JM Manz RA Henz BM Retinoic acid inhibits CD40 + interleukin-4-mediated IgE production in vitro. Blood. 1998; 92: 1713–1720. 9716600

[42] Xystrakis E Kusumakar S Boswell S Peek E Urry Z Richards DF Adikibi T Pridgeon C Dallman M Loke TK Robinson DS Barrat FJ O’Garra A Lavender P Lee TH Corrigan C Hawrylowicz CM Reversing the defective induction of IL-10-secreting regulatory T cells in glucocorticoid-resistant asthma patients. J Clin Invest. 2006; 116: 146–155. 16341266 10.1172/JCI21759 PMC1307558

[43] Zügel U Steinmeyer A Giesen C Asadullah K A novel immunosuppressive 1alpha,25-dihydroxyvitamin D3 analog with reduced hypercalcemic activity. J Invest Dermatol. 2002; 119: 1434–1442. 12485451 10.1046/j.1523-1747.2002.19623.x

